# Terminal complement inhibition in atypical haemolytic uremic syndrome: a single-centre experience

**DOI:** 10.3389/fphar.2025.1683188

**Published:** 2025-11-17

**Authors:** Valentin D. Mocanu, Bogdan M. Sorohan, Elena G. Micu, Sonia Bălănică, Bogdan Obrişcă, Roxana A. Jurubiță, Camelia A. Achim, Adrian C. Lungu, Cristina S. Căpuşă, Gabriel Mircescu, Gener Ismail

**Affiliations:** 1 Nephrology Department, “Carol Davila” University of Medicine and Pharmacy, Bucharest, Romania; 2 Department of Nephrology, Fundeni Clinical Institute, Bucharest, Romania; 3 Department of Nephrology, “Dr Carol Davila” Teaching Hospital of Nephrology, Bucharest, Romania

**Keywords:** atypical hemolytic uremic syndrome, thrombotic microangiopathy, complement inhibition, genetic mutations, end-stage kidney disease

## Abstract

**Background:**

Atypical hemolytic uremic syndrome (aHUS) is a rare thrombotic microangiopathy (TMA) caused by complement dysregulation, leading to microangiopathic anemia, thrombocytopenia, and acute kidney injury (AKI). Complement over activation typically results from genetic mutations in alternative pathway proteins. The genetic profile varies regionally and influences clinical phenotype and outcomes.

**Methods:**

We conducted a retrospective observational study in all 27 patients (12 children, 15 adults) diagnosed with aHUS, at “Fundeni” Clinical Institute, between January 2017 and January 2025. Median age was 30 years (range, 1–70), 59% female. All patients were treated with anti-C5 monoclonal antibodies and followed for a median of 13 months (9–27).

**Results:**

No patient had a family history of aHUS. Infections were the most common trigger (67%). Although 30% had a history of previous events, the median time from latest event to admission was 30 days, reflecting late diagnosis and referral, but the median time from admission to treatment was 8 days. At presentation, 60% of patients required dialysis and all had anemia, but 20% had no thrombocytopenia. AKI was common and the predominant clinical presentation was acute nephritic syndrome. Renal biopsies showed acute-on-chronic TMA with glomerulosclerosis and interstitial fibrosis in 90% and 80% of cases, respectively. Genetic testing revealed CFH/CFHR variants in 39% and CFI variants in 22% of patients. Anti-C5 therapy led to remission of anemia and thrombocytopenia in about 90% of patients, C3 normalization in 90%, and dialysis independence in 74%. No deaths or serious adverse events occurred.

**Conclusion:**

In this Romanian aHUS cohort, CFI variants were more frequent than expected, probably reflecting a different geographical distribution. Anti-C5 therapy proved effective and safe. However, limited patient numbers and observational design are study limitations.

## Introduction

aHUS is an extremely rare and often underdiagnosed condition, with an annual incidence estimated at 0.5–2 cases per million people (Liu and Qi, 2023). Timely diagnosis and intervention are crucial, as the disease carries a poor prognosis: up to 50% of patients progress to end-stage kidney disease (ESKD) within 10 years (Nester et al., 2015), and mortality ranges from 1% to 7% in the first year after onset (Fremeaux-Bacchi et al., 2013). aHUS affects all age groups, with equal sex distribution in children and a predominance in adult females (Goodship et al., 2017; Schaefer et al., 2018).

aHUS is a severe form of TMA, driven by uncontrolled activation of the alternative complement pathway. Diagnosis is based on the triad of AKI, microangiopathic anemia and thrombocytopenia, but requires exclusion of other TMAs—such as typical HUS and thrombotic thrombocytopenic purpura (TTP)—via stool cultures and ADAMTS13 activity testing (Goodship et al., 2017; Brocklebank et al., 2023; Brocklebank et al., 2018).

Extra-renal involvement occurs in 10%–30% of patients and may affect the neurological, cardiovascular, or gastrointestinal systems, often worsening prognosis. The disease course typically involves relapsing acute episodes that lead to progressive kidney damage (Schaefer et al., 2018; Loirat and Frémeaux-Bacchi, 2011).

Complement dysregulation results from genetic abnormalities in complement regulators—CFH, CFI, CFB, CD46, and C3—identified in 40%–60% of cases, with notable geographic variation. Autoantibodies against CFH, frequently associated with CFHR1/CFHR3 deletions, are also found in 6%–20% of patients (Schaefer et al., 2018; Brocklebank et al., 2018; Blanc et al., 2012; Józsi et al., 2008). The genetic burden is additive and is inversely corelated with the intervention of environmental trigger factors of acute aHUS events, which could unmask deficiency in alternative pathway regulation and explain the variable penetrance with age of genetic factors (Rodríguez de Córdoba, 2023).

Eculizumab, a monoclonal antibody anti-C5 improved aHUS prognosis to end stage kidney disease (ESKD) with over 70% and about 50% ESKD-free free survival at 1 and 5 years (Schaefer et al., 2018; Brocklebank et al., 2023; Legendre et al., 2013).

Given the rarity of aHUS and the potential for regional variability in genetic and environmental risk factors, national data are essential. Moreover, information on aHUS in Southeast Europe, including Romania, remains limited. Accordingly, this study aims to retrospectively analyse the clinical presentation, genetics and outcome in aHUS patients treated in a tertiary Romanian nephrology centre.

## Materials and methods

### Study design

We conducted a retrospective observational cohort study in all 27 patients with aHUS starting therapy with complement inhibitors (Eculizumab or Ravulizumab) between January 2017 and January 2025, in the nephrology (pediatric and adult) and transplant departments of the “Fundeni” Clinical Institute, Bucharest, Romania. Patients followed for less than 90 days were excluded.

TMA was suspected in case of association of AKI with thrombocytopenia and microangiopathic hemolytic anaemia. The diagnosis was confirmed by complement profiling, genetic analysis and, when possible, kidney biopsy. Secondary forms of TMA were excluded through relevant investigations, including ADAMTS13 activity (to rule out TTP), stool cultures for Shiga-like toxin-producing bacteria, and screening for autoimmune diseases, vasculitis, pregnancy, transplantation, and drug exposure.

### Data collection

Demographic data, family history, suspected triggers, clinical presentation, laboratory parameters (including complement, genetic and pathology findings) and treatment information were extracted from electronic medical records. Delays in diagnosis and treatment were assessed as follows: time from the most recent presumed aHUS-related event to hospital admission (diagnostic delay), and time from clinical suspicion to initiation of anti-complement therapy (treatment delay). Data were compared between admission and last follow-up.

### Laboratory data

Anemia was defined using WHO thresholds: Hb < 13 g/dL in men, <12 g/dL in women, <11 g/dL in women during pregnancy and in children. In cases of advanced CKD (eGFR <30 mL/min/1.73 m2) or kidney failure we accepted patients with a target Hb of 10.5-11.5 g/dL, if the blood smear was without schistocytes and there weren’t signs of hemolysis (normal LDH <250 U/mL and haptoglobin >0.3 g/dL). Thrombocytopenia was defined as platelet count <150.000*10^3^/L or a ≥25% drop from baseline. eGFR was calculated using the 2021 CKD-EPI equation. Complement components (C3 and soluble C5b-9) were measured by ELISA. Reference values were 90–180 mg/dL for C3 and 110–252 ng/mL for sC5b-9.

Kidney biopsies were performed in 10 patients and evaluated by light microscopy (toluidine blue), immunofluorescence (IgM, IgG, IgA, C3, C1q, κ/λ light chains, fibrinogen), and electron microscopy.

Genetic testing, performed in 23 patients, was conducted in collaboration with the Department of Internal Medicine and Haematology, Semmelweis University (Hungary). The protocol includes multiplex ligation-dependent probe amplification (MLPA) using the P236-A3 probe mix from MRC-Holland to identify deletions or duplications in the CFH, CFHR1, CFHR2, CFHR3, CFHR4, and CFHR5 genes. Additionally, direct DNA sequencing of PCR-amplified products from total genomic DNA was used to analyse the entire coding regions of several complement-related genes, including CFH, CFI, CD46, C3, CFB, THBD, and CFHR5.

### Anti-C5 therapy

Eculizumab was started empirically in suspected cases, with a switch to Ravulizumab when aHUS diagnosis was confirmed. Dosing followed protocols used in clinical trials, with weight-based adjustments (Legendre et al., 2013; Ariceta et al., 2021; Barbour et al., 2021). Adjuvant corticosteroids were administered when appropriate. All patients were vaccinated against *Neisseria meningitidis* and received antibiotic prophylaxis if treatment preceded immunization.

### Statistical analyses

Descriptive statistics for categorical variables were presented as frequencies and percentages. Continuous variables were summarized as medians and quartiles (q1; q3), as most investigated variables were non-normally distributed. Comparisons were done using Fisher exact test and Mann Witney or Willcoxon tests. A p value <0.05 was considered statistically significant. Statistical analyses were performed using the SPSS version 26 (IMB, NY, United States). Figures were created using GraphPad Prism version 10.0.0 (1992–2023 GraphPad Software, LLC, San Diego, CA, United States) and Biorender.

The study was conducted according to the Declaration of Helsinki and was approved by the Ethics Committee of”Fundeni” Clinical Institute (No. 15386).

## Results

### Clinical presentation

The median age was 30 years (range, 0–70); aHUS debuted in childhood in 44% of patients. There was a female predominance (59%). No patient had a family history of aHUS.

Infections were the predominant identified triggers, observed in two-thirds of patients, equally divided between respiratory and gastrointestinal causes (Table 1).

**TABLE 1:** Characteristics of aHUS patients at admission (N = 27).

Demographics	
Age, years (median)	30 (11–40)
Childhood onset	12 (44%)
Female	16 (59%)
Family history of aHUS	0 (0%)
Trigger factors	18 (67%)
Respiratory infections	9 (33%)
Gastroenteritis infections	9 (33%)
Multiple aHUS events before admission	8 (30%)
Delay in aHUS referral (days, median)	30 (9–92.5)
Delay in anti-complement therapy initiation (days, median)	8 (0.25–83.5)
Arterial hypertension	26 (96%)
Transfusions	13 (48.1%)
Adjuvant immunosuppression	14 (51.8%)
Kidney	
Renal syndrome	
AKI	24 (89%)
Acute nephritic syndrome	21 (78%)
Nephritic-nephrotic syndrome	2 (7%)
Chronic nephritic syndrome	4 (15%)
Hemodialysis	16 (59%)
Thrombotic microangiopathic anemia (n = 25)	18 (72%)
Microangiopathic anemia	23 (92%)
Thrombocytopenia	20 (80%)
AKI + thrombocytopenia + anemia	19 (76%)
Complement	
Low C3 (n = 25)	18 (72%)
High C5b-9 (n = 19)	14 (73%)

Data are presented as number (percent) or median (q1; q3); anemia was defined as Hb < 13 g/dL in men, <12 g/dL in females and <11 g/dL in children; thrombocytopenia was defined as <150.000*10^3^/L or by a >25% decrease from baseline; low C3 was defined as a value <90 mg/dL; high C5b-9 was defined as > 252 ng/mL.

The median time from the most recent acute aHUS event to referral was 30 days (range, 9–92.5), although about one-third of patients experienced multiple TMA events before admission. The median time from admission to anti-complement therapy initiation was 8 days (range, 0.25–83.5) (Table 1). The median time for eculizumab treatment was 9 months (range, 6–22) and ravulizumab was 4 months (range, 3–5).

At admission, all patients had proteinuria (median 1.95 g/day) and hematuria occurred in 96%. Serum creatinine was 3.41 mg/dL, corresponding to an eGFR 22 mL/min/1.73 m^2^. Acute nephritic syndrome (77%) was the main presentation, followed by chronic nephritic (15%) and nephrotic syndrome (7%) (Tables 1, 2).

**TABLE 2:** Laboratory data of patients of aHUS patients at admission and at last follow up (N = 27).

	Admission	Last follow up	P value
Kidney			
Serum creatinine (mg/dL)	3.41 (1.8–6.85)	1.18 (0.71–3.27)	0.012
eGFR (mL/min/1.73 m^2^)	22 (9.5–48.25)	80 (16–104.5)	0.001
Proteinuria (g/24 h)	1.95 (1.21–4.65)	0.36 (0.15–2.09)	0.046
Thrombotic microangiopathic anemia			
Hemoglobin (g/dL)	8.5 (6.8–9.85)	12.5 (10.55–13.2)	<0.001
LDH (u/L)	807.5 (488–1800)	224 (170–266)	<0.001
Haptoglobin (u/L)	0.1 (0.08–0.88)	1.18 (0.76–1.51)	0.001
Platelets (*10^3^/μL)	65 (27.5–207)	258 (206.5–346)	<0.001
Complement			
C3 (mg/dL)	73.2 (60.4–97.1)	103 (80.6–120.5)	0.001
C5b-9 (ng/mL)	362 (242–627)	184 (141–343)	0.019

Data are presented as number (%) or median (q1; q3); p-values were calculated using the Wilcoxon signed-rank test for paired comparisons; a p value of <0.05 was considered statistically significant; eGFR, estimated glomerular filtration rate; sCr - serum creatinine.

AKI was seen in 89% of cases, and was severe, stage III in 66%, imposed dialysis in 59% of patients. Microangiopathic anemia was present in 92% and thrombocytopenia in 80%. However, all three cardinal elements for TMA diagnosis were concomitantly present in 76% of cases. Median C3 was low (73.2 mg/dL) and was lower than the laboratory inferior limit in 72% of patients, as well as sC5b-9 was higher than the laboratory upper limit in 75% in patients with a median of 363 mg/dL (Tables 1, 2).

**FIGURE 1:**
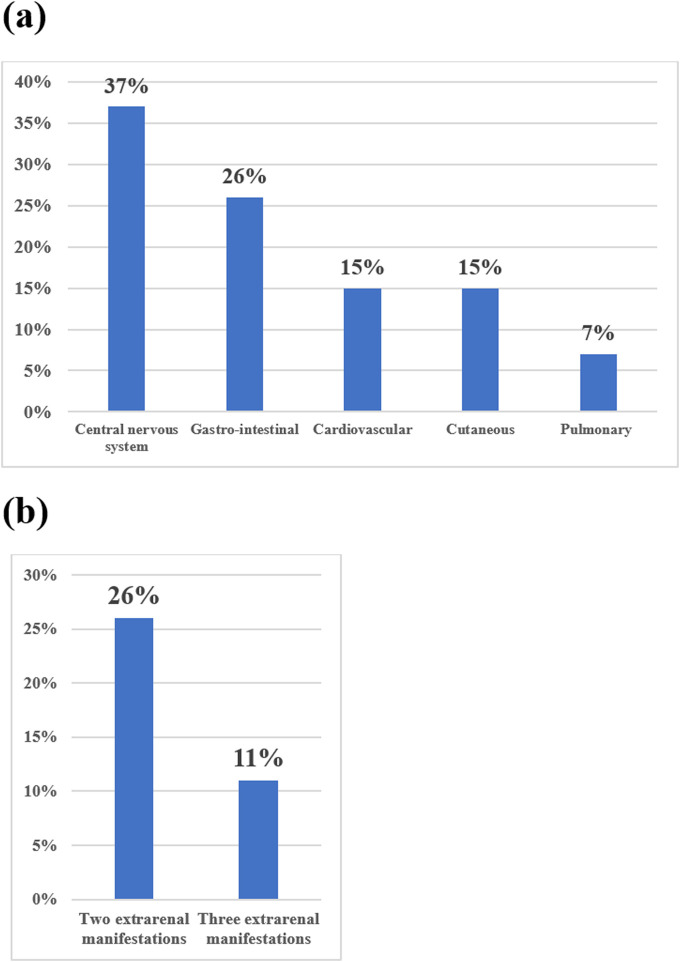
Extrarenal manifestations in aHUS acute phase (N = 27). **(a)** Extrarenal manifestations, ordered by frequency, highlight CNS involvement as most common (n = 10, 37%) followed by gastrointestinal (n = 7, 26%) cardiovascular/cutaneous (n = 4, 15% each), and pulmonary (n = 2, 7%); **(b)** 26% of patients had two affected systems simultaneously (n = 7), while 11% had three (n = 3).

All patients experienced at least one extrarenal manifestation, 26% had two and 11% had three extrarenal manifestations (Figure 1). The CNS manifestations were the most prevalent (37%) and had heterogeneous clinical presentation, ranging from confusion to delirium, to posterior reversible encephalopathy and coma. Gastrointestinal manifestations were seen in 25% of cases and acute pancreatitis was the most frequent. Cardiologic manifestations were seen in 15% of cases. Rupture of the tendinous chords, necessitating cardiac surgery, with favourable outcome occurred in one patient. Another patient developed acute-on-chronic heart failure secondary to malignant hypertension, but responded well to conservative management and three patients had left ventricular hypertrophy. Purpura was the only cutaneous manifestation in 15% patients. Pulmonary manifestations were rare (7%). Both patients presented with diffuse alveolar haemorrhage, one of whom received plasmapheresis alongside complement blockade.

### Genetic analyses

**FIGURE 2:**
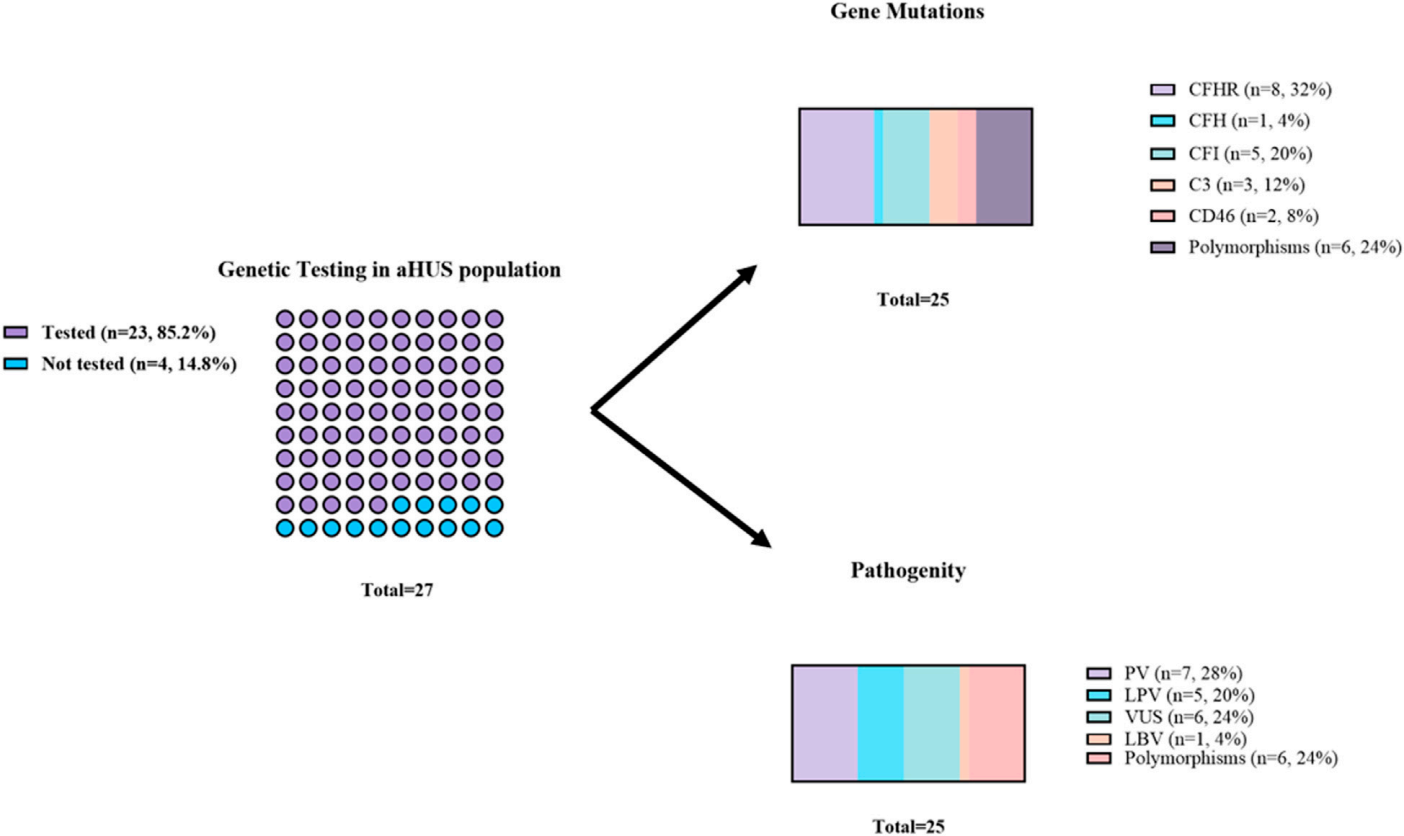
Gene mutations identified in aHUS patients. In the CFHR group were included all factor related proteins mutations. The mutation responsible for antoimmune aHUS (CFHR3/1 deletion were identified in 6 patients LPV Likely pathogenic variant, PV Pathogenic variant; VUS-variance of uncertain significance, LBV = likely benign version.

Genetic testing was performed in 23 out of 27 patients. Complement-related gene mutations were identified in 17 patients (74%). CFH and CFHR gene variants were analyzed together, given their shared genomic cluster (Dragon-Durey et al., 2008). Variants in CFH/CFHR genes were most frequent (35%), and CFHR3/1 deletions—often associated with anti-CFH autoantibodies—were observed in 26% of cases. Additional mutations were identified in CFI (22%), C3 (13%), and CD46 (9%) (Supplementary Table S1; Figure 2).

We fully characterised 7 new mutations: CD46*c.313 C>G, CFI*c.-13G>A, CFI*c.1278C>T, CFH*c.3592G>C, C3*c.3280G>A, C3*c.3804T>C and CFI*p.Gly292Val.

Only 30% of patients had pathogenic gene variants, and 26% had variants of unknown significance (VUS). However, based on patient history and clinical presentation, we suggest that another four VUS (#3 CD46*c.313 C>G; #4 CFI*c.-13G>A; #5 CFI*c.1278C>T; #16 C3*c.3804T>C), may be considered pathogenic variants (Supplementary Table S1).

Interestingly, 26% of patients in our cohort lacked pathogenic mutations, but carried 2–4 risk factors predisposing them to aHUS episodes upon exposure to other triggering events. We classified these patients under the term “combined mutations”. Compared to those with confirmed pathogenic mutations, this group exhibited a numerically older age, less thrombocytopenia and delayed referral and treatment initiation—though these differences were not statistically significant. However, the “combined mutations” group had significantly worse renal outcomes: a lower median eGFR at admission (10 vs. 36.5 mL/min/1.73 m^2^, p = 0.027), a more pronounced decline in eGFR at last follow-up (13 vs. 100 mL/min/1.73 m^2^, p = 0.001) and a lower proportion of patients remaining dialysis-free (50% vs. 94%, p = 0.004). These findings suggest that patients with multiple risk factors—even in the absence of pathogenic mutations—may experience more severe renal impairment than those with identified genetic variants (Table 3).

**TABLE 3:** Effects of combined mutations on aHUS clinical presentation and outcome.

	Gene mutation		p
	Pathogenic mutations	Combined mutations	
	n = 17	n = 6	
Demographics			
Age at Eculizumab initiation	19 (8–31)	42 (33.75–46.5)	0.116
Childhood onset (%)	12 (71%)	0 (0%)	0.005
Female (%)	13 (76%)	3 (50%)	0.537
Multiple TMA events before diagnosis (%)	9 (53%)	0 (0%)	0.34
Trigger factors (%)	12 (71%)	4 (67%)	1
Delay in aHUS referral, days (median)	16 (6.75–53.25)	51 (11.5–93.5)	0.721
Delay in anticomplement therapy initiation, days (median)	1 (0–39.25)	59 (7.25–241.5)	0.127
Kidney			
AKI stage III at admission (%)	11 (65%)	4 (67%)	1
eGFR at admission (median, mL/min/1.73 m^2^)	36.5 (14–56.5)	10 (6.5–20.25)	0.027
eGFR last follow-up (median, mL/min/1.73 m^2^)	100 (81.5–116)	13 (7.25–33)	0.001
Hemodialysis			
Before admission (%)	5 (29%)	0 (0%)	0.272
At the first admission	10 (59%)	4 (67%)	1
During follow up	1 (6%)	1 (17%)	0.04
Hemodialysis-free at last follow-up (%)	16 (94%)	3 (50%)	0.04
Thrombotic microangiopathic anemia			
Microangiopathic anemia at baseline (%)	13 (76%)	5 (83%)	1
Hemoglobin at admission (median, g/dL)	8.6 (6.4–9.2)	9.3 (7.6–10.3)	0.353
Hemoglobin at last follow-up (median, g/dL)	12.6 (12–13.5)	12.3 (10.5–12.5)	0.34
Thrombocytopenia at admission	13 (76%)	3 (50%)	0.089
Thrombocytes at admission (median, *1,000/mma^3^)	57 (25–136.5)	137.5 (36.7–279.5)	0.445
Thrombocytes at last follow-up (median, *1,000/mm^3^)	293 (232.5–346)	256 (208.7–370)	0.850
Anemia + thrombocytopenia at admission	14 (82%)	3 (50%)	0.279
AKI + Anemia + thrombocytopenia at admission	14 (82%)	3 (50%)	0.279
Complement			
C3 at admission (median, mg/dL)	70 (53–80)	90.6 (58.1–105)	0.381
C3 at last follow-up (median, mg/dL)	99 (76.2–105.5)	121 (90.3–128.7)	0.154
MAC at admission (median, mg/dL)	396.5 (227.5–728)	355 (296.7–431.25)	0.820
MAC at last follow-up (median, mg/dL)	254.5 (173.2–361.2)	171.6 (136.7–212.4)	0.154

Anemia was defined as Hb < 13 g/dL in men, <12 g/dL in females and <11 g/dL in children; thrombocytopenia was defined as <150.000*10^3^/L or by a >25% decrease from baseline; we used Mann-Whitney U test for continuous variables and Fischer’s exact test for all categorical variables.

### Kidney biopsy

aHUS is primarily a clinical diagnosis and kidney biopsy is often unsafe because of thrombocytopenia and high bleeding risk. In our centre, renal biopsy was performed only when histology was expected to influence diagnosis or management. The main indications were atypical presentations such as chronic nephritic syndrome or slowly progressive CKD (unexplained decline in eGFR >50% over 2–5 years, with or without cytopenias, where the differential diagnosis included other glomerular diseases, for obtaining prognostic information regarding recovery of the kidney function or for the suspected recurrence in a kidney allograft. Ten patients underwent kidney biopsy, that mainly presented with acute nephritic syndrome for 60% of them and chronic nephritic syndrome in the other 40% at the time of this procedure. Most showed TMA on light microscopy; two had ischemic glomerulopathy and one had membranoproliferative with acute TMA lesions and crescents.

Acute glomerular capillary TMA lesions, endotheliosis, endothelial swelling, but not thrombi, were observed, while chronic changes - double contours, hyaline deposits, glomerulosclerosis, and tubulointerstitial fibrosis - were common. Onion-skin arteriopathy was rare (1 patient). Acute-on-chronic lesions were more frequent (6/10) than purely acute lesions (4/10).

Podocytes effacement was seen in all cases, but endothelial proliferation was observed only in the patient with MPGN pattern, where dense deposits were also present. In this case, C3 bright staining suggested C3 glomerulopathy (Supplementary Table S2).

### Outcome

#### Kidney

Following anti-complement therapy, kidney function improved rapidly. Proteinuria decreased from 1.95 to 0.36 g/24 h. Partial (≥25% eGFR increase from baseline) and complete (eGFR >60 mL/min/1.73 m^2^) renal recovery were observed in 26% and 61% of patients, occurring after median times of 7.5 days (4.25; 27.5) and 20 days (Schaefer et al., 2018; Fakhouri et al., 2017), respectively. Among patients initially on dialysis, 70% (9/13) became dialysis-free within a median of 19.5 days (10; 73). At last follow-up, 74% were dialysis-independent (Tables 2, 4).

**TABLE 4:** Outcomes of aHUS patients under anti-C5 therapy (N = 27**)**.

	N = 27
Referral delay (days, median)	30 (9–92.5)
Treatment initiation delay (days, median)	8 (0.25–83.5)
Kidney	
Recovery of kidney function	
complete (eGFR >60 mL/min/1.7 m^2^) (%)	14 (61%)
days to complete renal remission (median)	20 (5; 31)
partial (eGFR ≥25% from baseline) (%)	6 (26%)
days to partial renal remission (median)	7.5 (4.25–27.5)
Hemodialysis	
withdrawal (%)	9 (69%)*
days to hemodialysis withdrawal (median)	19.5 (10–73)
hemodialysis free at last follow-up (%)	20 (74%)
Kidney transplantation (%)	2 (8%)
Anemia	
Complete remission	23 (85%)
Days to increase in Hb ≥ 25% from baseline (median)	32 (8.5–56)
Days to complete remission (median)	78 (47.5–119.25)
Thrombocytopenia	
complete remission (>150*10^3^/μL) (%)	25 (93%)
Days to a ≥25% increase from baseline (median)	3 (2–4)
Days to complete remission (median)	8 (4–32.5)
Complement	
Days to C3 normalization (>90 mg/dL) (median)	51 (15–79)
Days to C5b-c9 normalization (< 252 ng/mL) (median)	153.5 (49–920.5)

*of those on hemodialsis during admission (n = 15).

Data are presented as number (%) or median (q1; q3).

eGFR, estimated glomerular filtration rate; ESKD, end stage kidney disease; Hb–hemoglobulin.

**FIGURE 3:**
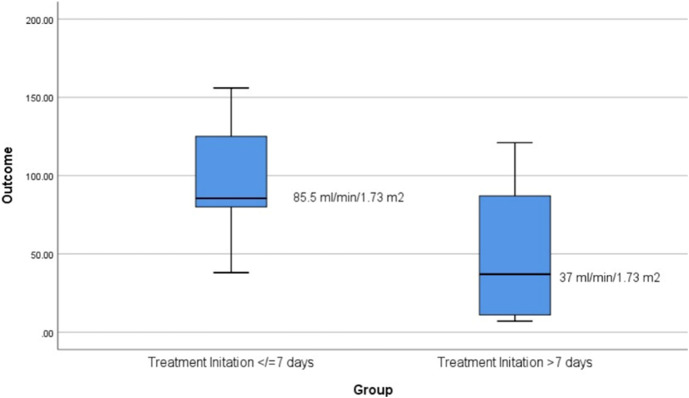
Box plot of eGFR by treatment initiation timing: Early vs. Late. The median eGFR was significantly higher in patients who initiated treatment within 7 days (85.5 mL/min/1.73 m², IOR: 71.0–126.25) compared to those who started treatment after 7 days (37.0 mL/min/1.73 m², IOR: 10.25–90.25). The Mann-Whitney U test indicated a statistically significant difference between the two groups (U = 22.00, Z = −2.117.p = 0.034).

Earlier therapy initiation was associated with better renal outcomes. Median eGFR was significantly higher in patients treated within 7 days (85.5 vs. 37.0 mL/min/1.73 m^2^, p < 0.05) (Figure 3). Two patients received kidney transplants—one received Eculizumab before and one after transplantation—and both achieved complete functional recovery. The latter had an acute aHUS flare with pathologically confirmed TMA kidney lesions.

Of the 8 patients on dialysis at the last follow-up, four were already on dialysis because of delayed diagnosis, resulting in advanced chronic kidney lesions with low eGFR (around 20 mL/min/1.73 m^2^) at anti-complement therapy initiation.

#### Thrombocytopenic microangiopathic anemia

Microangiopathic anemia resolved in 85% of patients. Partial remission occurred by day 32 and full remission by day 78. About half required blood transfusions. Hemolysis markers (LDH, haptoglobin) normalized at last follow-up (Table 2; Supplementary Table S1). Thrombocytopenia resolved in 93% of cases (25/27). Among the two patients with persistent thrombocytopenia, one belonged to the combined mutations group and one had not been genetically tested. Platelet counts began improving by day 3 and normalized by day 8 post-treatment. Four patients (15%) did not achieve complete resolution of anemia: one carried a pathogenic variant, two had not been genetically tested, and one belonged to the combined mutations group. Thus, incomplete hematologic remission was not confined to a specific genetic category.

#### Complement

At last follow-up, low C3 levels normalized (103 mg/dL; 80.6–120.5 mg/dL), while elevated C5b-9 levels decreased (184 mg/dL; 141–343 mg/dL). It is noteworthy that, even at baseline, 28% of patients already had C3 levels >90 mg/dL. This subgroup was genetically heterogeneous (3 patients with genetically proven aHUS, 2 with combined mutations and 2 were not tested). Thus, having a normal C3 concentration at presentation was not restricted to patients without a complement gene defect, indicating that baseline C3 values do not necessarily correlate with the presence or absence of a pathogenic variant.

#### Adverse effects

No relapses, deaths, or severe adverse events occurred during treatment, supporting the safety and effectiveness of anti-C5 therapy in this cohort.

## Discussion

To our knowledge, this is the first report on aHUS patients diagnosed and treated with anti-C5 complement inhibitors in Romania. Study results highlight some characteristics particular to our geographical region: a high time to diagnosis, preponderance of CNS events in the acute phase, chronic kidney lesions consecutive to frequent previous acute events and a higher than reported frequency of CFI genes mutations. The therapy with anti-C5 monoclonal antibodies was efficient and safe. However, improvement can be obtained by increasing physicians’ awareness for aHUS diagnosis.

### Clinical presentation

Our cohort had a median age of 30 years, the youngest being 1 year-old and the oldest, 70 years-old. Thus, as described in other cohorts, aHUS could affect individuals of all ages, despite the strong genetic background (gene variants are reported in 40%-60% of cases), which supports the role of trigger factors, commonly infections, pregnancy, surgery and transplantation, which unravel deficiencies in alternative pathway regulation (Schaefer et al., 2018; Brocklebank et al., 2023; Spasiano et al., 2023). Family history, reported in other studies up to 16%, were absent in this cohort, possibly due to geographical variation, but infections triggered acute aHUS events in 67% of cases, aligning with findings from other cohorts (62%–100%) (Tomazos et al., 2020; Piras et al., 2022). *De novo* or relapsing aHUS cases were described after vaccination (Bouwmeester et al., 2022) and we documented an episode of TMA triggered by influenza vaccination.

Previous aHUS events were present in higher proportion in our patients as compared to other cohorts (30% vs.10%–20%). The high rate of previous aHUS events could result in a more frequent acute-on-chronic aHUS course as previously described (Loirat and Frémeaux-Bacchi, 2011) also in majority of patients from our cohort. This is further reinforced by clinical presentations of our patients such as chronic nephritic or nephrotic syndrome and biopsy findings of acute-on-chronic TMA lesions. There is precedent in the literature for clinically inapparent or subclinical TMA episodes in patients with complement dysregulation and other causes of TMA. Penetrance of pathogenic complement variants is incomplete and many events appear to require a transient trigger; because of that, episodes of endothelial injury/TMA can be focal or transient and may resolve without long-term, specific therapy, yet leave chronic tubular and glomerular lesions on biopsy (Loirat and Frémeaux-Bacchi, 2011; Fujino et al., 2025).

At presentation, AKI and microangiopathic anemia were observed in about 90% of cases, but thrombocytopenia was less frequent (80%). All three components of aHUS diagnostic triad were present in 76%, similar to reported in the French study (74%) (Fremeaux-Bacchi et al., 2013), emphasizing that diagnosis remains challenging in a subset of patients.

Kidney biopsy is useful to orient diagnosis to aHUS by identifying TMA lesions, when aHUS is difficult to diagnose because of the smouldering course. On the other hand, in case of an acute-on-chronic presentation, biopsy can provide prognostic information (Gallan and Chang, 2020; Kim, 2022). In our cohort, acute-on-chronic pattern of aHUS was more frequent and tubulointerstitial fibrosis was prominent, in line with patients’ history and clinical presentation. Podocytes effacement was common, explaining nephrotic proteinuria observed in some patients and highlighting the role of ischemia and activated complement in podocytes dysfunction (Noris et al., 2015).

In the acute aHUS phase, a quarter of patients had extrarenal manifestations simultaneously affecting two organs. Unlike data from the International aHUS Registry (IaHUSR), in our cohort, CNS, not gastrointestinal manifestations were the most frequent, and cardiovascular manifestations were on the third place instead of the second^2^. These data highlight the clinical complexity of acute aHUS phase and possibly reflect genetic and zonal variations (Loirat and Frémeaux-Bacchi, 2011; Brocklebank et al., 2020).

The role of C3 and sC5b-9 levels in aHUS diagnosis remains uncertain (Bu et al., 2015). In our study, 25% of patients had C3 and sC5b-9 values within normal limits at presentation, limiting their diagnostic utility. Nevertheless, a strong clinical suspicion remains essential for early diagnosis, particularly when laboratory data are inconclusive.

### Genetic mutations

About 74% of tested patients had mutations in genes coding complement proteins, a proportion higher than reported in other studies (40%–60%) (Schaefer et al., 2018; Brocklebank et al., 2018). However, when only pathogenic and likely pathogenic variants were considered, this proportion decreased to 52%.

Our study also fully characterized seven new potential risk allele in CD46, CFI, CFH and C3 genes, and suggested reclassification as pathologic of five VUS, but this should be confirmed in other studies.

**FIGURE 4:**
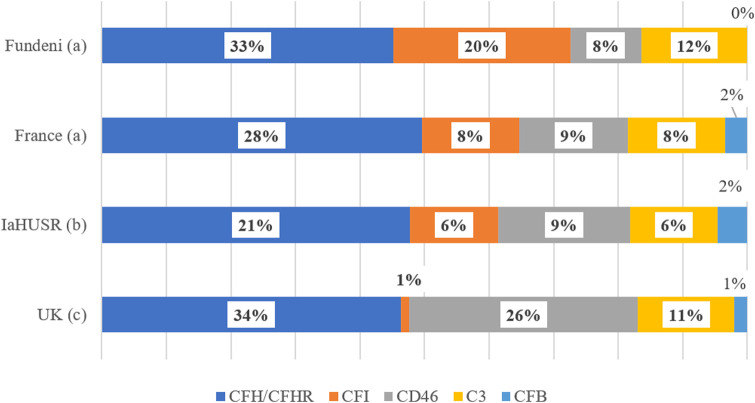
Share of mutation identified in “Fundeni” Institute (“Fundeni”), French, International aHUS Registry (IaHUSR) and United Kingdom (United Kingdom) cohort. (a) all patients with gene mutation identified; (b) all patients tested; (c) all patients with gene mutation and anti-CFH antibodies. Data from (Fremeaux-Bacchi et al., 2013; Brocklebank et al., 2018; Schaefer et al., 2018).

Mutations in the CFH/CFHR gene cluster were the most frequent, observed in 39% of tested individuals. CFI mutations were identified in 22% of patients, followed by C3 (13%) and CD46 (9%). While the distribution of CFH, CD46, and C3 mutations aligns with data from the IaHUSR, the United Kingdom, and France, CFI variants were notably more common in our cohort (22% vs. 1%–8%) (Fremeaux-Bacchi et al., 2013; Schaefer et al., 2018; Brocklebank et al., 2018) (Figure 4).

Geographical differences in distribution of complement-related genes variants were also described in other studies, for example, CFH mutations predominate in China (Wu et al., 2021), whereas C3 mutations are more common in Japan (Fujisawa et al., 2018). Accordingly, the increased frequency of CFI variants observed in our study may reflect a region-specific genetic pattern, warranting further investigation in broader Romanian or Southeast European cohorts.

CFHR3/1 deletions were the most frequent abnormal variants observed in our cohort and the proportion was higher than reported (26% vs. 16%) (Zipfel et al., 2007). This deletion was associate with anti-CFH auto-antibodies blocking CFH activity and “immune” aHUS (9,10,15). CFHR3/1 deletions were also related to infections acting as trigger factor, and in our cohort, infections were seen in 67% of cases. A recent study described a relation between CFH and CFHR structural variants and infection, suggesting that dysfunctional CFH could not adequately control the complement activation induced by exposure to pathogens (Piras et al., 2022).

Genetic abnormalities also appeared to influence disease severity. Variants in CFH, CFI, and C3 have previously been associated with higher ESKD risk, while CD46 mutations tend to confer a more favorable prognosis (Schaefer et al., 2018; Brocklebank et al., 2018; Piras et al., 2022). However, our cohort was not large enough and the number of patients reaching ESKD was too low to test this association.

Combined mutations, defined as the presence of risk factors in more than one complement gene, are frequent in aHUS (24% of tested patients in our cohort) and could also influence prognosis. Moreover, multiple combined mutations could explain the variable penetrance of genetic mutations (Rodríguez de Córdoba, 2023; Bresin et al., 2013; Fakhouri et al., 2017).

### Outcome

In this study, 60% of patients on dialysis became dialysis-free in about 3 weeks, 61% of patients achieved a complete kidney function remission and 74% were dialysis-free at last follow-up. Additionally, Eculizumab therapy improved kidney graft outcome in two transplanted patients with acute aHUS. These good results are in line with previous reports (over 70% chances of dialysis-free survival in the first year), supporting the efficiency of anti-complement agents in aHUS (Fremeaux-Bacchi et al., 2013; Brocklebank et al., 2023; Legendre et al., 2013; Al Riyami et al., 2023; Fakhouri et al., 2016).

Importantly, early initiation of therapy was a key predictor of renal outcome. In our cohort, starting treatment within 7 days of clinical suspicion was associated with significantly better eGFR at follow-up (85.5 vs. 37.0 mL/min/1.73 m^2^, p = 0.034), consistent with findings from other studies (Legendre et al., 2013; Tatematsu et al., 2024). However, in the IaHUSR cohort, the time from the most recent aHUS event to presentation was not independently associated to kidney outcome (Schaefer et al., 2018), which suggests that other factors than early therapy initiation, like pre-existent chronic kidney lesions, also play a significant role in recovery potential.

Our study revealed that patients within the combined mutations group experienced significantly worse renal outcomes compared to those with confirmed genetic variants. This group exhibited a lower median eGFR at admission (10 vs. 36.5 mL/min/1.73 m^2^, p = 0.027), more severe renal impairment at follow-up (13 vs. 100 mL/min/1.73 m^2^, p = 0.001) and a markedly reduced proportion of dialysis-free survival (50% vs. 94%, p = 0.004). Clinically, these patients tended to be older, with less pronounced thrombocytopenia and complement consumption, no prior TMA episodes, and delayed diagnosis and treatment initiation—though these differences did not reach statistical significance, possibly due to our cohort’s limited size and follow-up duration. Notably, their clinical presentation appeared less ‘typical’ of classical aHUS, which may contribute to diagnostic delays and suboptimal early management. These findings align with previous studies (Brocklebank et al., 2023; Bresin et al., 2013), suggesting that this group may represent a distinct clinical phenotype. Further studies in larger cohorts are needed to validate these observations and clarify the underlying mechanisms.

Before 2022, access to Eculizumab in Romania was limited to compassionate use, contributing to treatment delays.

Hematologic recovery was also favourable. Anemia improved in 85% of patients (complete remission at 78 days), while thrombocytopenia resolved more rapidly, with platelet counts normalizing by day 8. Markers of hemolysis normalized by last follow-up.

Eculizumab and Ravulizumab efficiently increased C3 and decreased sC5b-9 plasma levels after 51 and 154 days.

No relapses, deaths, or severe infections—including meningococcal disease—were reported, supporting the safety of anti-C5 therapy.

This study has several limitations. It was a single-centre retrospective study, with a low number of participants and a short period of observation, resulting in a less consistent statistical support and a limited generalization to other populations. However, this study summarizes the largest clinical experience in aHUS management in our country and highlights both deficiencies of care to be improved and the need of a national aHUS registry.

## Conclusion

We provided for the first time data on clinical presentation, genetic profiles, and outcomes in aHUS patients treated with anti-C5 monoclonal antibodies in Romania. Our findings suggest a relatively long delay in aHUS diagnosis and potential genetic difference in distribution of genes variants within the Romanian population, with CFI mutations more frequently observed. Anti-C5 therapy was effective and safe. Our data suggest creation of a National aHUS Registry and of a standardized protocol of management, and increased physicians’ awareness to improve aHUS care in our country.

## Data Availability

The original contributions presented in the study are included in the article/Supplementary Material, further inquiries can be directed to the corresponding author.

## References

[B1] Al Riyami D. Mohammed S. Al Salmi I. Metry A. Al Kalbani N. Almurshadi F. (2023). Epidemiology, management, and outcome of atypical hemolytic uremic syndrome in an Omani cohort. Oman Med. J. 38 (6), e569. 10.5001/omj.2023.117 38317858 PMC10839635

[B2] Ariceta G. Dixon B. P. Kim S. H. Kapur G. Mauch T. Ortiz S. (2021). The long-acting C5 inhibitor, ravulizumab, is effective and safe in pediatric patients with atypical hemolytic uremic syndrome naïve to complement inhibitor treatment. Kidney Int. 100 (1), 225–237. 10.1016/j.kint.2020.10.046 33307104

[B3] Barbour T. Scully M. Ariceta G. Cataland S. Garlo K. Heyne N. (2021). Long-term efficacy and safety of the long-acting complement C5 inhibitor ravulizumab for the treatment of atypical hemolytic uremic syndrome in adults. Kidney Int. Rep. 6 (6), 1603–1613. 10.1016/j.ekir.2021.03.884 34169200 PMC8207473

[B4] Blanc C. Roumenina L. T. Ashraf Y. Hyvärinen S. Sethi S. K. Ranchin B. (2012). Overall neutralization of complement factor H by autoantibodies in the acute phase of the autoimmune form of atypical hemolytic uremic syndrome. J. Immunol. 189, 3528–3537. 10.4049/jimmunol.1200679 22922817

[B5] Bouwmeester R. N. Bormans E. M. G. Duineveld C. van Zuilen A. D. van de Logt A. E. Wetzels J. F. M. (2022). COVID-19 vaccination and atypical hemolytic uremic syndrome. Front. Immunol. 13, 1056153. 10.3389/fimmu.2022.1056153 36531998 PMC9755835

[B6] Bresin E. Rurali E. Caprioli J. Sanchez-Corral P. Fremeaux-Bacchi V. Rodriguez de Cordoba S. (2013). Combined complement gene mutations in atypical hemolytic uremic syndrome influence clinical phenotype. J. Am. Soc. Nephrol. 24 (3), 475–486. 10.1681/ASN.2012090884 23431077 PMC3582207

[B7] Brocklebank V. Wood K. M. Kavanagh D. (2018). Thrombotic microangiopathy and the kidney. Clin. J. Am. Soc. Nephrol. 13 (2), 300–317. 10.2215/CJN.00620117 29042465 PMC5967417

[B8] Brocklebank V. Kumar G. Howie A. J. Chandar J. Milford D. V. Craze J. (2020). Long-term outcomes and response to treatment in diacylglycerol kinase epsilon nephropathy. Kidney Int. 97 (6), 1260–1274. 10.1016/j.kint.2020.01.045 32386968 PMC7242908

[B9] Brocklebank V. Walsh P. R. Smith-Jackson K. Hallam T. M. Marchbank K. J. Wilson V. (2023). Atypical hemolytic uremic syndrome in the era of terminal complement inhibition: an observational cohort study. Blood 142 (16), 1371–1386. 10.1182/blood.2022018833 37369098 PMC10651868

[B10] Bu F. Meyer N. C. Zhang Y. Borsa N. G. Thomas C. Nester C. (2015). Soluble c5b-9 as a biomarker for complement activation in atypical hemolytic uremic syndrome. Am. J. Kidney Dis. 65 (6), 968–969. 10.1053/j.ajkd.2015.02.326 25818678

[B12] Dragon-Durey M. A. Blanc C. Marliot F. Loirat C. Blouin J. Sautes-Fridman C. (2008). The high frequency of complement factor H related CFHR1 gene deletion is restricted to specific subgroups of patients with atypical haemolytic uraemic syndrome. J. Med. Genet. 46, 447–450. 10.1136/jmg.2008.064766 19435718

[B13] Fakhouri F. Hourmant M. Campistol J. M. Cataland S. R. Espinosa M. Gaber A. O. (2016). Terminal complement inhibitor eculizumab in adult patients with atypical hemolytic uremic syndrome: a single-arm open-label trial. Am. J. Kidney Dis. 68, 84–93. 10.1053/j.ajkd.2015.12.034 27012908

[B14] Fakhouri F. Zuber J. Frémeaux-Bacchi V. Loirat C. (2017). Haemolytic uraemic syndrome. Lancet. 390 (10095), 681–696. 10.1016/S0140-6736(17)30062-4 28242109

[B15] Fremeaux-Bacchi V. Fakhouri F. Garnier A. Bienaimé F. Dragon-Durey M. A. Ngo S. (2013). Genetics and outcome of atypical hemolytic uremic syndrome: a nationwide French series comparing children and adults. Clin. J. Am. Soc. Nephrol. 8 (4), 554–562. 10.2215/CJN.04760512 23307876 PMC3613948

[B16] Fujino Y. Hanada H. Takata T. Kageyama K. Taniguchi S. Mae Y. (2025). C3 p.Asp1115Asn variant-associated atypical hemolytic uremic syndrome with spontaneous remission triggered by influenza and COVID-19. Intern Med. 11, 6036-25. 10.2169/internalmedicine.6036-25 40930830 PMC13175673

[B17] Fujisawa M. Kato H. Yoshida Y. Usui T. Takata M. Fujimoto M. (2018). Clinical characteristics and genetic backgrounds of Japanese patients with atypical hemolytic uremic syndrome. Clin. Exp. Nephrol. 22 (5), 1088–1099. 10.1007/s10157-018-1549-3 29511899 PMC6437120

[B18] Gallan A. J. Chang A. (2020). A new paradigm for renal thrombotic microangiopathy. Seminars Diagnostic Pathology 37 (3), 121–126. 10.1053/j.semdp.2020.01.002 32085935

[B19] Goodship T. H. J. Cook H. T. Fakhouri F. Fervenza F. C. Frémeaux-Bacchi V. Kavanagh D. (2017). Atypical hemolytic uremic syndrome and C3 glomerulopathy: conclusions from a “Kidney Disease: improving Global Outcomes” (KDIGO) controversies conference. Kidney Int. 91 (3), 539–551. 10.1016/j.kint.2016.10.005 27989322

[B20] Józsi M. Licht C. Strobel S. Zipfel S. L. H. Richter H. Heinen S. (2008). Factor H autoantibodies in atypical hemolytic uremic syndrome correlate with CFHR1/CFHR3 deficiency. Blood 111 (3), 1512–1514. 10.1182/blood-2007-09-109876 18006700

[B21] Kim Y. J. (2022). A new pathological perspective on thrombotic microangiopathy. Kidney Res. Clin. Pract. 41 (5), 524–532. 10.23876/j.krcp.22.010 35791743 PMC9576460

[B22] Legendre C. M. Licht C. Muus P. Greenbaum L. A. Babu S. Bedrosian C. (2013). Terminal complement inhibitor eculizumab in atypical hemolytic-uremic syndrome. N. Engl. J. Med. 368 (23), 2169–2181. 10.1056/NEJMoa1208981 23738544

[B23] Liu Q. Qi H. M. (2023). Evolution in the diagnosis and treatment of hemolytic uremic syndrome. Asian J. Surg. 46 (2), 919–921. 10.1016/j.asjsur.2022.07.055 35963672

[B24] Loirat C. Frémeaux-Bacchi V. (2011). Atypical hemolytic uremic syndrome. Orphanet J. Rare Dis. 6, 60. 10.1186/1750-1172-6-60 21902819 PMC3198674

[B25] Nester C. M. Barbour T. de Cordoba S. R. Dragon-Durey M. A. Fremeaux-Bacchi V. Goodship T. H. J. (2015). Atypical aHUS: state of the art. Mol. Immunol. 67, 31–42. 10.1016/j.molimm.2015.03.246 25843230

[B26] Noris M. Mele C. Remuzzi G. (2015). Podocyte dysfunction in atypical haemolytic uraemic syndrome. Nat. Rev. Nephrol. 11 (4), 245–252. 10.1038/nrneph.2014.250 25599621

[B27] Piras R. Valoti E. Alberti M. Bresin E. Mele C. Breno M. (2022). CFH and CFHR structural variants in atypical hemolytic uremic syndrome: prevalence, genomic characterization and impact on outcome. Front. Immunol. 13, 1011580. 10.3389/fimmu.2022.1011580 36793547 PMC9923232

[B28] Rodríguez de Córdoba S. (2023). Genetic variability shapes the alternative pathway complement activity and predisposition to complement-related diseases. Immunol. Rev. 313 (1), 71–90. 10.1111/imr.13131 36089777 PMC10086816

[B29] Schaefer F. Ardissino G. Ariceta G. Fakhouri F. Scully M. Isbel N. (2018). Clinical and genetic predictors of atypical hemolytic uremic syndrome phenotype and outcome. Kidney Int. 94 (2), 408–418. 10.1016/j.kint.2018.02.029 29907460

[B30] Spasiano A. Palazzetti D. Dimartino L. Bruno F. Baccaro R. Pesce F. (2023). Underlying genetics of aHUS: which connection with outcome and treatment discontinuation? Int. J. Mol. Sci. 24, 14496. 10.3390/ijms241914496 37833944 PMC10572301

[B31] Tatematsu Y. Imaizumi T. Michihata N. Kato N. Kumazawa R. Matsui H. (2024). Annual trends in atypical haemolytic uremic syndrome management in Japan and factors influencing early diagnosis and treatment: a retrospective study. Sci. Rep. 14 (1), 18265. 10.1038/s41598-024-68736-6 39107421 PMC11303750

[B32] Tomazos I. Garlo K. Wang Y. Chen P. Laurence J. (2020). Triggers in patients with atypical hemolytic uremic syndrome: an observational cohort study using a US claims database. Blood 136, 30–31. 10.1182/blood-2020-136278

[B33] Wu D. Chen J. Ling C. Chen Z. Fan J. Sun Q. (2021). Clinical and genetic characteristics of atypical hemolytic uremic syndrome in children: a Chinese cohort study. Nephron 145 (4), 415–427. 10.1159/000513009 33873197

[B34] Zipfel P. F. Edey M. Heinen S. Józsi M. Richter H. Misselwitz J. (2007). Deletion of complement factor H-related genes CFHR1 and CFHR3 is associated with atypical hemolytic uremic syndrome. PLoS Genet. 3 (3), e41. 10.1371/journal.pgen.0030041 17367211 PMC1828695

