# Correction: Terminal complement inhibition in atypical haemolytic uremic syndrome: a single-centre experience

**DOI:** 10.3389/fphar.2025.1760223

**Published:** 2025-12-11

**Authors:** Valentin D. Mocanu, Bogdan M. Sorohan, Elena G. Micu, Sonia Bălănică, Bogdan Obrişcă, Roxana A. Jurubiță, Camelia A. Achim, Adrian C. Lungu, Cristina S. Căpuşă, Gabriel Mircescu, Gener Ismail

**Affiliations:** 1 Nephrology Department, “Carol Davila” University of Medicine and Pharmacy, Bucharest, Romania; 2 Department of Nephrology, Fundeni Clinical Institute, Bucharest, Romania; 3 Department of Nephrology, “Dr Carol Davila” Teaching Hospital of Nephrology, Bucharest, Romania

**Keywords:** atypical hemolytic uremic syndrome, thrombotic microangiopathy, complement inhibition, genetic mutations, end-stage kidney disease

An incorrect **Funding statement** was provided. The correct funding statement reads:

The author(s) declare that financial support was received for the research and/or publication of this article. Publication of this paper was supported by the University of Medicine and Pharmacy Carol Davila, through the institutional program Publish not Perish.

The original article has been updated.

