# Supplementary material for: Terminal complement inhibition in atypical haemolytic uremic syndrome: a single-centre experience
Source: Front Pharmacol. 2025 Nov 17;16:1683188. doi: 10.3389/fphar.2025.1683188 (PMC12665522; doi:10.3389/fphar.2025.1683188)
Supplement: Supplementary file 1 [file Table1.docx]

Supplementary Table 1. Genetic abnormalities in patients with aHUS (N=23)

| 1 | 2 | 3 | 4 | 5 | 6 | 7 | 8 | 9 | 10 |
| --- | --- | --- | --- | --- | --- | --- | --- | --- | --- |
| No | **Gene** | **Identified mutation** | **Consequence** | **Zygosity** | **Novel mutation** | **Pathogenicity** | **FH** | **CD 46** | **C3** |
| 1 | CFHR | Deletion in CFHR3/1 | Anti-CFH Ab | HOM | No | PV | 2 | 1 | 2 |
| 2 | CFHR | Deletion in CFHR3/1 | Anti-CFH Ab | HOM | No | PV | 0 | 0 | 0 |
| 3 | CD46 | c.313 C>G | Pro105Ala | HET | Yes | VUS | 0 | 0 | 0 |
| 4 | CFI | c.-13G>A | 5'UTR | HET | Yes | VUS | 2 | 0 | 0 |
| 6 | CFI | c.1278C>T | p.Y426Y | HET | Yes | VUS | 2 | 2 | 2 |
| 7 | CFHR | Deletion in CFHR3/1 | Anti-CFH Ab | HOM | No | PV | 0 | 1 | 2 |
| 8 | Combined mutations | No pathogenic mutation | Risk Factor |  |  |  | 1 | 1 | 2 |
| 9 | CFH | c.3592G>C | p.E1198Q | HET | Yes | LPV | 3 | 1 | 0 |
|  | CFHR5 | c.1067G>A | p.R356H | HET | No | VUS |  |  |  |
| 10 | CFI | c.782G>A | Gly261Asp | HET | No | LPV | 2 | 1 | 0 |
| 11 | CFI | c.215C>G | p.T72S | HET | No | LPV | 1 | 1 | 0 |
| 12 | C3 | c.3280G>A | Ala1094Thr | HET | Yes | LPV | 1 | 1 | 0 |
| 13 | Combined mutations | No pathogenic mutation | Risk Factor |  |  |  | 2 | 1 | 1 |
| 14 | Combined mutations | No pathogenic mutation | Risk Factor |  |  |  | 2 | 1 | 0 |
| 15 | Combined mutations | No pathogenic mutation | Risk Factor |  |  |  | 2 | 0 | 2 |
| 16 | C3 | c.3804T>C | p.S1268S | HET | Yes | VUS | 2 | 1 | 2 |
| 18 | Combined mutations | No pathogenic mutation | Risk Factor |  |  |  | 2 | 1 | 0 |
| 19 | CFHR | Deletion in CFHR3/1 | Anti-CFH Ab | HOM | No | PV | 1 | 0 | 0 |
| 20 | CD46 | No pathogenic mutation | p.Tyr189Asp | HET | No | PV | 1 | 1 | 0 |
| 21 | CFHR | Deletion in CFHR3/1 | Anti-CFH Ab | HOM | No | PV | 0 | 0 | 0 |
| 22 | CFHR | Deletion in CFHR3/1 | Anti-CFH Ab | HOM | No | PV | 0 | 1 | 0 |
| 23 | CFI | CFIc.875G>T | p.Gly292Val |  | Yes | LPV | 2 | 1 | 0 |
|  | CFHR | Deletion in CFHR1,3,4 | Probably a large deletion of exon 6 of CFHR3, CFHR1 gene and part of CFHR4 | HET | No | VUS |  |  |  |
| 24 | Combined mutations | No pathogenic mutation | Risk Factor |  |  |  | 0 | 1 | 1 |
| 25 | C3 | c.3125G>A | Arg1042Gln | HET | No | LPV | 2 | 1 | 2 |
| CFHR = complement factor H related protein; Ab = antibodies; HOM = homozygous; HET = heterozygous CFH = complement factor H; CFI = complement factor I; LPV - Likely pathogenic variant; PV - Pathogenic variant; VUS – Variance of uncertain significance; in the #2, #3 and #4 columns we showed the gene implicated with the nucleotide change (#3) and the protein change (#4); we used the term combined mutations for additional genetic risk factors (without pathogenic mutations) that may precipitate a thrombotic microangiopathy (TMA) episode; in columns #8, 9 and 10 there are represented additional risk factors for developing atypical hemolytic uremic syndrome in the factor H, CD 46 and C3. | | | | | | | | | |

**Supplementary table 2. Histopathological characteristics of patients that underwent kidney biopsy**

| **Patient No** | **4** | **5** | **6** | **7** | **8** | **14** | **15** | **17** | **26** | **27** |
| --- | --- | --- | --- | --- | --- | --- | --- | --- | --- | --- |
| **Main histological feature** | **TMA** | **TMA** | **Ischemic glomerulopathy** | **TMA** | **TMA** | **Ischemic glomerulopathy** | **TMA** | **MPGN + cellular crescents** | **TMA** | **TMA** |
| **Thrombi in GC** | 0 | 0 | 0 | 1 | 0 | 0 | 0 | 0 | 0 | 0 |
| **Arteriolar thrombi** | 0 | 0 | 0 | 0 | 0 | 0 | 1 | 0 | 0 | 0 |
| **Glomeruli/sclerosed** | 2/2 | 3/3 | 6/1 | 3/2 | 9/1 | 2/1 | 5/4 | 18/10 | 5/0 | 11/6 |
| **Percent** | 100% | 100% | 16.66% | 66.66% | 11.11% | 50% | 80% | 55.55% | 0% | 54.54% |
| **Endocapillary proliferation** | 0 | 0 | 0 | 0 | 0 | 0 | 0 | 1 | 0 | 1 |
| **Double contour** | 1 | 1 | 0 | 0 | 1 | 0 | 1 | 1 | 1 | 0 |
| **Mesangial hypertrophy** | 0 | 0 | 1 | 0 | 0 | 1 | 0 | 1 | 0 | 0 |
| **Onion skin** | 0 | 1 | 0 | 0 | 0 | 0 | 0 | 0 | 0 | 0 |
| **Crescents** | 0 | 0 | 0 | 0 | 0 | 0 | 0 | 2 | 0 | 1 |
| **IFTA** | 1 | 2 | 0 | 3 | 1 | 1 | 3 | 0 | 1 | 2 |
| **ATN** | 0 | 0 | 0 | 0 | 0 | 0 | 0 | 0 | 0 | 0 |
| **Interstitial inflammation** | 1 | 1 | 0 | 1 | 1 | 1 | 1 | 1 | 1 | 1 |
| **Arteriolosclerosis** | 1 | 1 | 0 | 1 | 0 | 0 | 1 | 0 | 1 | 0 |
| **Endotheliosis** | 1 | 0 | 0 | 1 | 1 | 1 | 1 | 0 | 1 | NA |
| **Endothelial swelling** | 1 | 1 | 0 | 0 | 1 | 0 | 0 | 1 | 1 | 1 |
| **Widening of the subendothelial area** | 1 | 1 | 0 | 0 | 1 | 0 | 0 | 1 | 1 | 0 |
| **Hyaline deposits** | 0 | 1 | 1 | 0 |  | 0 | 1 | 1 | 1 | 0 |
| **IF** | C3 | Negative | 0 | 0 | 0 | NA | Fibrin | C3>IgA, IgG, L>K | C3, IgM, K, L | 0 |
| **Podocytes effacement** | 2 | 1 | 1 | 2 |  | 1 | 2 | 1 | 1 | NA |
| **Dense deposits** | 0 | 0 | 0 | 0 | 0 | 0 | 0 | 1 | 0 | NA |
| *GC = glomerular capillaries, TMA = thrombotic microangiopathy, MPGN = membranoproliferative glomerulonephritis, IFTA = interstitial fibrosis and tubular atrophy, ATN = acute tubular necrosis, IF = immunofluorescence; for crescents we used 0 for their absence, 1 for less than 50% of the affected glomeruli and 2 for more than 50% of affected glomeruli; for IFTA we used 0 if there were < 10%, 1 for 10-25%, 2 for 25-50% and 3 for more than 50% of the affected tubulointerstitial compartment* | | | | | | | | | | |
